# Genetic analysis of 20 patients with hypomyelinating leukodystrophy by trio-based whole-exome sequencing

**DOI:** 10.1038/s10038-020-00896-5

**Published:** 2021-02-18

**Authors:** Huifang Yan, Haoran Ji, Thomas Kubisiak, Ye Wu, Jiangxi Xiao, Qiang Gu, Yanling Yang, Han Xie, Taoyun Ji, Kai Gao, Dongxiao Li, Hui Xiong, Zhen Shi, Ming Li, Yuehua Zhang, Ruoyu Duan, Xinhua Bao, Yuwu Jiang, Margit Burmeister, Jingmin Wang

**Affiliations:** 1 Department of Pediatrics, Peking University First Hospital, Beijing, China; 2 Molecular & Behavioral Neuroscience Institute, University of Michigan, Ann Arbor, MI USA; 3 Joint International Research Center of Translational and Clinical Research, Beijing, China; 4 Department of Radiology, Peking University First Hospital, Beijing, China; 5 Key Laboratory for Neuroscience, Ministry of Education/National Health and Family Planning Commission, Peking University, Beijing, China; 6 Departments of Computational Medicine & Bioinformatics, Psychiatry and Human Genetics, University of Michigan, Ann Arbor, MI USA

## Abstract

Hypomyelinating leukodystrophies (HLDs) are a rare group of disorders characterized by myelin deficit of the brain-based on MRI. Here, we studied 20 patients with unexplained HLD to uncover their genetic etiology through whole-exome sequencing (WES). Trio-based WES was performed for 20 unresolved HLDs families after genetic tests for the PLP1 duplication and a panel of 115 known leukodystrophy-related genes. Variants in both known genes that related to HLDs and promising candidate genes were analyzed. Minigene splicing assay was conducted to confirm the effect of splice region variant. All 20 patients were diagnosed with HLDs clinically based on myelin deficit on MRI and impaired motor ability. Through WES, in 11 of 20 trios, 15 causative variants were detected in seven genes *TUBB4A, POLR1C, POLR3A*, *SOX10*, *TMEM106B, DEGS1*, and *TMEM63A*. The last three genes have just been discovered. Of 15 variants, six were novel. Using minigene splicing assay, splice variant *POLR3A* c.1770 + 5 G > C was proved to disrupt the normal splicing of intron 13 and led to a premature stop codon at position 618 (p.(P591Vfs*28)). Our analysis determined the molecular diagnosis of 11 HLDs patients. It emphasizes the heterogenicity of HLDs, the diagnostic power of trio-based WES for HLDs. Comprehensive analysis including a focus on candidate genes helps to discover novel disease-causing genes, determine the diagnosis for the first time, and improve the yield of WES. Moreover, novel mutations identified in *TUBB4A, POLR3A*, and *POLR1C* expand the mutation spectrum of these genes.

## Introduction

Hypomyelinating leukodystrophies (HLDs) are a specific group of leukodystrophies characterized by an unchanged pattern of deficient myelination on MRI scans at least 6 months apart in a child between the ages of 1–2 years [1, 2]. HLDs are genetically and clinically diverse, but have a prominent commonality: motor ability is affected in most HLDs patients, often as ataxia with cerebellar signs. In addition, variable intellectual disability can also be observed [1, 3]. The prototype of HLD1 is Pelizaeus–Merzbacher disease (PMD [MIM: 312080]) due to variants in the myelin protein proteolipid protein 1 (*PLP1* [MIM: 300401]). Before we started our research, 15 disease-causing genes related to HLDs, have been defined in OMIM.

In our HLDs cohort in China, out of 205 cases, genetic tests for the PLP1 duplication and a panel of 115 known leukodystrophy-related genes diagnostically solved 155 cases (~75%). In this study, 20 pedigrees, from the remaining 50 unresolved cases, underwent trio-based whole-exome sequencing (WES) to uncover their genetic etiology.

## Materials and methods

### Undiagnosed patients

Twenty core pedigrees in this study were a portion of our HLDs cohort with 205 cases clinically diagnosed with HLDs, collected at Peking University First Hospital (Beijing, China). To elucidate the genetic causes, *PLP1* dosage was initially examined using multiplex ligation-dependent probe amplification (MLPA) to exclude *PLP1* duplication, the most common cause of HLD1. Subsequently, variants in 115 leukodystrophy-related genes (see Online Resource 1) were examined by targeted next-generation sequencing (NGS) (Kangso Medical Inspection, China).

Of 205 families, pathogenic variants in 13 known HLDs genes were identified in 75.6% (155/205 of the patients 119 cases have been published [4]). For the remaining 50 unresolved families, 20 core pedigrees were enrolled for this study according to the availability of DNA samples of index patients and their parents. Informed consent was obtained from the patients’ guardians and approved by the Medical Ethics Committee of Peking University First Hospital.

### Whole-exome sequencing (WES)

**Fig. 1:**
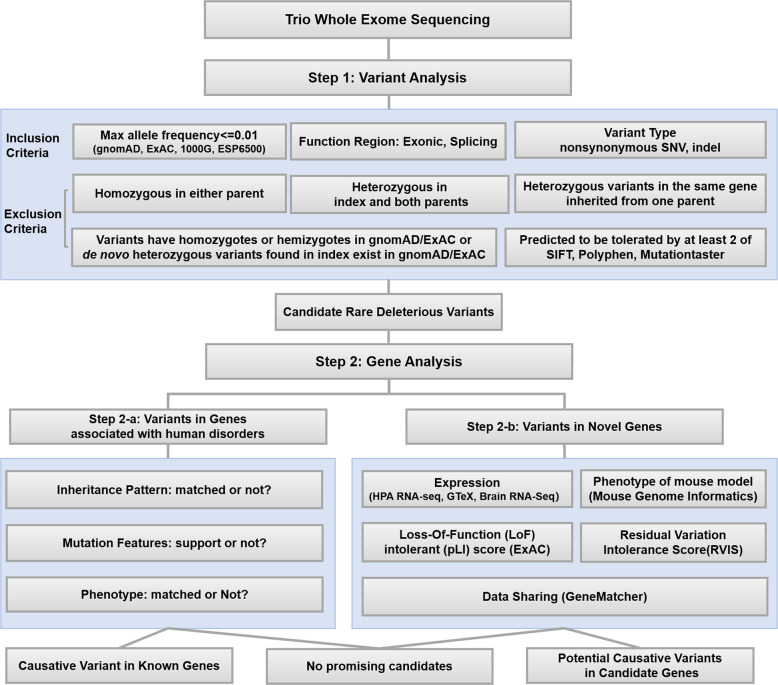
In-house analysis workflow of trio-based exome sequencing data. gnomAD genome aggregation database, ExAC exome aggregation consortium, 1000G 1000 genomes project, ESP6500, exome sequencing project

Exons were captured by SeqCap EZ MedExome Kit (Roche NimblenGen) and SureSelect Human All Exon V6 (Agilent, US) followed by sequencing on an Illumina X10 (2 × 150-nucleotide paired-end reads) by Joy Orient Translational Medicine Research Center Co., Ltd. Company (Beijing, China) and Wuxi NextCODE Genomics (Shanghai, China) Co., Ltd., respectively. Reads were aligned to the UCSC human reference genome (hg19) build using the Burrows-Wheeler aligner [5]. Variants were called using GATK HaplotypeCaller v3.7 and were annotated by variant effect predictor [6]. For the splice variant, two different prediction tools human splicing finder (HSF, http://www.umd.be/HSF3/) [7] and splice site prediction (SSP, http://www.fruitfly.org/seq_tools/splice.html) were used to estimate the variant’s impact on the transcript. For each patient, variants were filtered and prioritized according to the in-house analytical workflow (Fig. 1) and classified into three categories: (i) causative variants in known genes; (ii) potentially causative variants in candidate genes; or (iii) no promising candidates. All candidate variants were confirmed by Sanger sequencing.

The data that support the findings of this study are available on request from the corresponding author. The data are not publicly available due to privacy and ethical restrictions.

### Minigene splicing assay

In vitro, a minigene splicing assay was performed as described before [8]. Briefly, the genomic DNA of the patient with *POLR3A* c.1770 + 5 G > C variant and his mother as control were amplified. The products including exon 13, intron 13, exon 14, and the 5′ and 3′ flanking introns of *POLR3A* were ligated in the pcDNA3.1 and pEGFP-C1 plasmids. Constructs were sequenced by Sanger. Mutant and wild-type vectors were transfected into HCE and HEK-293T cell lines, respectively. Total RNA was harvested 36 h post-transfection and complementary DNA (cDNA) was transcribed using random primers. PCR was performed using cDNA and primers specific to the 5′ and 3′ native exons of the pcDNA3.1 and pEGFP-C1 vectors. The size and sequence of products were analyzed by agarose gel and Sanger.

## Results

Of 20 patients (Pt1–Pt20), 14 were male and 6 were female. The median age was 4 years 1 month (range from 10 months to 11 years). Variable developmental delay in motor ability and hypomyelination was observed in all 20 subjects (100%), and nystagmus in 17 patients (85%). Delayed cognitive ability was also a common feature occurring in 15 patients (75%). Developmental regression, ataxia, and other brain abnormalities such as atrophy or abnormal signals in the cerebellum and basal ganglia manifested in 4/20 (20%), 5/20 (25%), and 5/20 (25%) of patients, respectively. Abnormal visual evoked potentials (4/7, 57%) and brain-stem auditory evoked potential (7/10, 70%) were also worth noting (Table 1).

**Table 1:** Clinical features of all 20 patients

Pt	Gender	Age	Motor delay	Intelligence delay	Nystagmus	Regression	Ataxia	MRI	Mutated Gene
1	Female	2y3m	+++	+++	√	−	−	H, ACB, BG	*TUBB4A*
2	Male	6y3m	+++	++	√	√	√	H, BG	*TUBB4A*
3	Male	4y11m	++++	++	√	−	√	H, BG	*TUBB4A*
4	Male	9y7m	+	−	√	√	√	H, BS	*POLR3A*
5	Male	3y4m	+	−	√	√	√	H	*POLR1C*
6	Male	11y	+	+	√	√	√	H, ACB	*POLR1C*
7	Female	1y4m	+	+	−	−	−	H	*SOX10*
8	Female	3y4m	+	−	√	−	−	H	*TMEM106B*
9	Female	5y1m	++	+	−	−	−	H	*DEGS1*
10	Female	5y6m	+	−	√	−	−	H	*TMEM63A*
11	Male	4y2m	+++	+++	√	−	−	H^a^	*TMEM63A*
12	Male	4y7m	+	+	√	−	−	H, SCB	
13	Male	4y6m	++++	++++	√	−	−	H, BS	
14	Male	1y9m	+++	+	√	−	−	H	−
15	Male	4y5m	+++	+++	√	−	−	H	−
16	Female	1y3m	+	+	√	−	−	H,	−
17	Male	10m	++	−	√	−	−	H	−
18	Male	1y1m	++	+	√	−	−	H	−
19	Male	11m	+	+	√	−	−	H	−
20	Male	1y3m	+++	+++	−	−	−	H	−

√ positive, − negative, + mild delay, ++ moderate delay, +++ severe delay, ++++ profound delay, *m* months, *y* years, *H* hypomyelination, *ACB* atrophy of cerebellum, *SCB* abnormal signal in cerebellum to abnormal signal in cerebellar peduncle and white matter, *BS* abnormal signal in brain stem, *BG* abnormal signal and atrophy of basal ganglia

^a^Indicated that hypomyelination in Patient 11 resolved over time

Out of 20 trios, seven (Pt1–Pt7) (35%) presented with causative variants in four previous known HLDs genes before our study *TUBB4A* (MIM: 602662)*, POLR1C* (MIM: 610060)*, POLR3A* (MIM: 614258), and *SOX10* (MIM: 602229). Four (Pt8–Pt11) (30%) harbored potential causative variants in three candidate genes *TMEM106B* (MIM: 613413)*, DEGS1* (MIM: 615843), and *TMEM63A*, which were confirmed to be novel disease-causing genes of HLDs in subsequent studies [9–12]. No positive results were observed in the remaining nine trios (Pt12–Pt20).

The variant spectrum of all 15 standing out variants included 11 missense variants, 1 nonsense variant, 2 small in-frame deletion variants, and 1 splice region variant. All of them are rare in the published population: three in genes with recessive inheritance pattern with a low frequency (≤0.000116), while the remaining 12 variants are absent from 1000 genomes project (1000G), exome sequencing project (ESP6500), exome aggregation consortium (ExAC) or genome aggregation database (gnomAD). All missense and nonsense variants were predicted to be deleterious based on multiple prediction tools including SIFT, PolyPhen, MutationTaster, Condel, M-CAP, and PROVEAN (Table 2). For two in-frame insertion/deletion variants, *POLR3A* c.661_662insCCT (p.(P220_L221insS)) and *POLR1C* c.883_885delAAG (p.(K295del)), considering deleted residues were highly conserved and located in the functional domain and nonrepeat regions of the relative protein, both were predicted to be deleterious.

**Table 2:** Genetic characteristics of 20 HLDs patients

Pt	Gene	Transcript	CDS	Protein	Or	N/R	GE	CA	SI	Po	MT	Co	MC	PR
1	*TUBB4A*	NM_006087.3	c.1164 G > T	p.(M388I)	d	N	3.4	23.8	D	D	D	D	N	D
2	*TUBB4A*	NM_006087.3	c.1062 C > G	p.(C354W)	d	R	0.6	25.5	D	D	D	D	D	D
3	*TUBB4A*	NM_006087.3	c.1062 C > G	p.(C354W)	d	R	0.6	25.5	D	D	D	D	D	D
4	*POLR3A*	NM_007055.3	c.661_662insCCT	p.(P220_L221insS)	m	N	5.1	–	–	–	–	–	–	–
			c.1770 + 5 G > C	p.(P591Vfs*28)	p	N	4.8	7.2	–	–	–	–	–	-–
5	*POLR1C*	NM_001318876.1	c.322 C > T	p.(H108Y)	p	N	5.6	29.8	D	D	D	D	D	D
			c.883_885delAAG	p.(K295del)	m	R	4.9	14.6	–	–	–	–	–	–
6	*POLR1C*	NM_001318876.1	c.326 G > A	p.(R109H)	p	R	4.8	34.0	D	D	D	D	D	D
			c.901 C > T	p.(R301W)	m	N	4.9	35.0	D	D	D	D	D	D
7	*SOX10*	NM_006941.3	c.227 T > A	p.(V76D)	d	N	4.4	27.6	D	D	D	D	D	D
8	*TMEM106B*	NM_001134232.1	c.754 G > A	p.(D252N)	d	#	5.4	32.0	D	D	D	D	D	D
9	*DEGS1*	NM_001321541.1	c.110 T > C	p.(M37T)	p	#	5.5	24.6	D	D	D	D	D	D
			c.770 G > A	p.(W257*)	m	#	5.9	43.0	–	–	D	–	–	–
10	*TMEM63A*	NM_014698.2	c.1385 T > A	p.(I462N)	d	#	5.1	31.0	D	D	D	D	D	D
11	*TMEM63A*	NM_014698.2	c.503 G > A	p.(G168E)	d	#	5.9	26.9	D	D	D	D	D	D

*Pt* patient, *Or* origin, *m* maternal, *p* paternal, *d* de novo, *N* novel, *R* reported, *GE* GERP + + RS, CA CADD, *SI* SIFT, *Po* PolyPhen, *MT* mutation taster, *Co* Condel, *MC* M-CAP, PR PROVEAN, *D* probably damaging in polyphen or deleterious in other software, *N* neutral, # patients with these variants have been reported in our previous paper

**Fig. 2:**
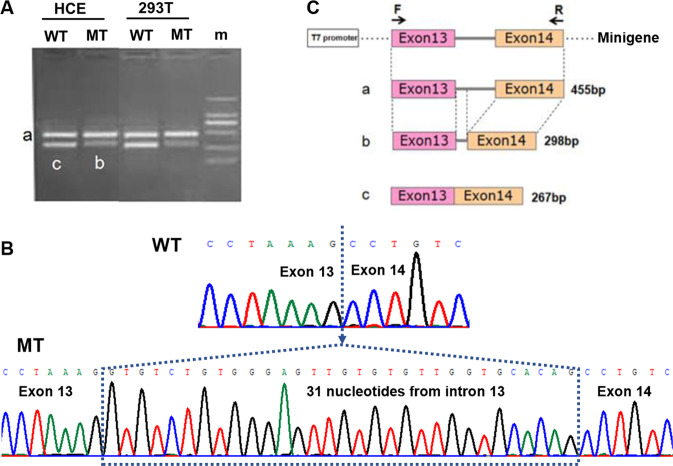
Minigene results of *POLR3A* c.1770 + 5 G > C variant. **a** Agarose gel image of PCR products. **b** Sanger sequence results of the PCR products. **c** Schematic of the minigene and aberrant splicing. WT wild type, MT mutant, m marker

For the splice region variant *POLR3A* c.1770 + 5 G > C, with a consensus value of −13.48% in HSF and SSP’s prediction score dropping from 0.97 to 0, both programs strongly predicted that the variant disrupts the wild-type donor site and a new donor splice site at c.1770 + 32_1770 + 33GT is generated with a SSP’s score of 0.90. This predicted aberrant splicing will change the reading frame, leads to the retention of 31 nucleotides from intron 13, and creates a premature stop codon at position 618 (p.(P591Vfs*28)). Minigene splicing assay in HCE and HEK-293T cells further confirm the change (Fig. 2). Of 15 variants, six have not been reported before and seven arose de novo.

### Causative variants in previously known genes

**Fig. 3:**
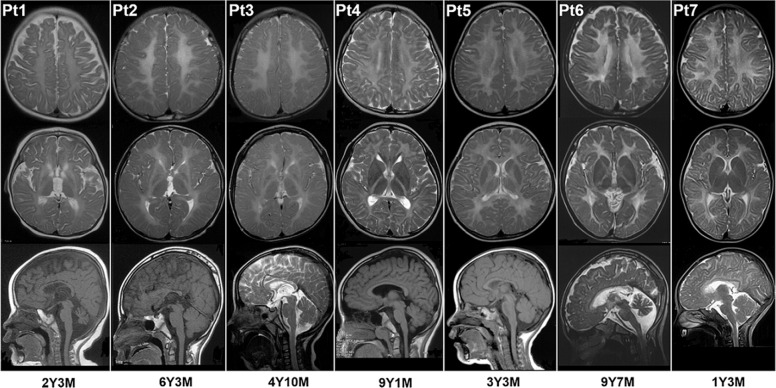
MRI images of seven patients. Row 1 and row 2 are axial T2-weighted images, showing diffuse hypomyelination in cerebral white matter in all seven patients and atrophy of the basal ganglia in Pt1, Pt2, and Pt3. Pt1 also demonstrated an abnormal signal in the putamen and caudate nucleus. Raw 3 are midsagittal T1-weighted or T2-weighted images, showing atrophy of cerebellum in Pt1 and Pt6. Six patients were diagnosed with HLD6 (Pt1, Pt2, Pt3), HLD7 (Pt4), HLD11 (Pt5, Pt6), and SOX10 related disorder (Pt7), respectively. Y years, M months

Unrelated Pt1, Pt2, and Pt3 harbored de novo heterozygous variants in *TUBB4A*, variants in which lead to HLD6, also named as hypomyelination with atrophy of the basal ganglia and cerebellum (H-ABC, [MIM: 612438]). The three variants were missed by clinical targeted NGS analysis due to miss-alignment and quality control filters. Pt1’s phenotype, including severe developmental delay, nystagmus, hypertonia, and diffused hypomyelination, the abnormal signal in the putamen and caudate nucleus, and progressive atrophy of the basal ganglia and cerebellum on MRI (Fig. 3), were highly concordant with classical H-ABC. Although presented with the same variants, the phenotypes of Pt2 and Pt3 were slightly different, and their phenotypes were somewhat atypical. In addition to development delay and nystagmus, motor regression onset at age of 2 years drew Pt2 parents’ attention and progressively worsens to a spastic gait. Microcephaly was also noted. MRI performed at age of 1 year and 9 months and at 6 years and 3 months showed hypomyelination and progressive atrophy of the basal ganglia without cerebellar involvement (Fig. 3). Clinical symptoms of Pt3 included development delay, nystagmus, and ataxia, without development regression and microcephaly. His MRI findings were consistent with Pt2 (Fig. 3).

Compound heterozygous variants in *POLR3A* and *POLR1C* were detected in Pt4, Pt5, and Pt6, respectively. Bi-allelic variants in *POLR3A* and *POLR1C* result in recessive HLD7 (MIM: 607694) and HLD11 (MIM:616494), respectively. Both are referred to as POLR3-related leukodystrophies [13]. Consistent with POLR3-related leukodystrophies, motor dysfunction, and diffused cerebral hypomyelination was present in all three patients (Fig. 3). Myopia and cerebellar atrophy were observed only in Pt6.

A de novo heterozygous missense variant in *SOX10* was discovered in Pt7. The phenotype of Pt7 included mild developmental delay and diffuse hypomyelination on MRI (Fig. 3). No other obvious clinical symptoms presented.

### Potential causative variant in three “candidate genes”

Deleterious variants in three genes *TMEM106B, DEGS1*, and *TMEM63A* were identified in Pt8–Pt11, respectively. In the early stage of the study, all these three genes had not been associated with HLD. We defined them as “candidate genes” based on the following evidence (Table 3). All three genes are expressed in the human brain [14] and oligodendrocytes [15], suggesting they may be involved in myelination. In silico analysis, they all have fewer reported variants than expected with positive missense *Z* scores [0.38, 0.35, 0.24], indicating increased constraint (intolerance to variation) [16]. *DEGS1* is predicted to be with a high probability of being loss-of-function (LoF) intolerant (pLI) with a relatively high pLI score of 0.86 [16]. In addition, the residual variation intolerance score (RVIS) of three genes, a measure (ranking) of intolerance, are relatively low [0.3 (35.46%), −0.08 (45.36%), −0.14 (42.37%)], which suggest that they are intolerant to functional genetic variation [17]. Based on the mouse genome informatics database (MGI) (http://www.informatics.jax.org/), tremors and abnormal gait were observed in *Degs1* and *Tmem63a* null mice, respectively, which are consistent with the phenotype of HLDs patient. Multiple systems including, growth, metabolism, and reproductive system, were affected in *Tmem106b* null mice. The genes were put on the data-sharing platform GeneMatcher [18], tracked dynamically, and were confirmed to be novel disease-causing genes eventually within two years by us and another group [9–12]. The corresponding phenotypes were named as HLD16 (MIM:617964), HLD18 (MIM: 618404), and HLD19 (MIM: 618688), respectively, in OMIM.

**Table 3:** Features of three candidate genes

Gene	Expression (RPKM)		Missense *Z* score	pLI	RVIS	Neurological symptom in null mice
	Brain	Oligodendrocyte				
*TMEM106B*	18.12	8.01	0.38	0.52	−0.3 (35.46%)	Tremor
*DEGS1*	20.699	14.91	0.35	0.86	−0.08 (45.36%)	Abnormal gait
*TMEM63A*	11.931	6.15	0.24	0	−0.14 (42.37%)	Not noted

The expression data was obtained from HPA RNA-seq normal tissues project (https://www.ncbi.nlm.nih.gov/bioproject/PRJEB4337/) and brain RNA seq database (http://www.brainrnaseq.org/). Missense Z and pLI score were from ExAC database (http://exac.broadinstitute.org/). RVIS came from the genetic intolerance database (http://genic-intolerance.org/). Phenotype of the mouse model was from mouse genome informatics (MGI) (http://www.informatics.jax.org/)

*RPKM* reads per kilobase per million mapped reads, *pLI* probability of being loss-of-function intolerant, *RVIS* residual variation intolerance score

## Discussion

In this study, we analyzed the clinical and genetic features of patients with hypomyelinating leukodystrophy. As suspected, hypomyelination on MRI and damaged motor ability due to the deficit of the myelin are essential symptoms of all 20 HLDs patients during infancy. Nystagmus and delayed cognitive ability are prevalent in most patients. Regression, ataxia, and other brain abnormalities were observed in a few patients. It is difficult to diagnose based only on clinical results. The comprehensive genetic investigation is useful for the molecular diagnosis of HLDs. In our HLDs cohort, genetic tests including MLPA targeted for the PLP1 duplication and a panel of 115 known leukodystrophy-related genes diagnostically solved ~75% of cases (155/205). Here, trio-based WES helps to confirm the molecular diagnosis of another 11 pedigrees, who were diagnosed with HLD6 (MIM: 612438), HLD7 (MIM: 607694), HLD11 (MIM: 616494), SOX10 related disorder, HLD16 (MIM: 617964), HLD18 (MIM: 618404) and HLD19 (MIM: 618688), respectively. Significant heterogenicity of HLDs was emphasized.

Since patients in this study had been screened for HLD1 and other 114 types of leukodystrophies, the diagnostic yield of WES is beyond our expectation. Studies of WES in HLDs have been reported by Vanderver et al. in 2016 [19]. Out of 71 patients with unresolved leukodystrophy, 35% (25/71) presented with diagnostic variants, 7% (5/71) had potential pathogenic variants in clinically relevant genes. The total yield of clinical diagnoses was 42% [19]. Similarly, Arai-Ichinoi et al. discovered the causative variant in known HLDs genes in 35% (6/17) of patients with unexplained hypomyelination using WES [20]. The unexpected diagnostic yield in this study is attributed to our focus on candidate genes and dynamic tracking. It helps to increase diagnosis yield from 35 to 55%. Our study demonstrates that identification of candidate genes in the initial analysis and tracking them dynamically help to discover novel disease-causing genes and determine the diagnosis for the first time, and WES is a powerful tool for Mendelian disease gene discovery.

Of 15 causative variants, six have not been reported before (*TUBB4A* c.1164 G > T p.(M388I), *POLR3A* c.661_662insCCT p.(P220_L221insS), *POLR3A* c.1770 + 5 G > C p.(P591Vfs*28), *POLR1C* c.322 C > T p.(H108Y), *POLR1C* c.901 C > T p.(R301W), and *SOX10* c.227 T > A), which expand the mutation spectrum of these genes. Mutation at position M388 in *TUBB4A* with different nucleotide changes, c.1162 A > G p.(M388V), c.1163 T > C p.(M388T), c.1164 G > A p.(M388I), have been reported before [21]. Mutations in intron 13 of *POLR3A* including 1771-7 C > G and c.1771-6 C > G have been reported, but their accurate effect on splicing is not verified by experiment [22, 23]. Here, using minigene splicing assay, splice variant *POLR3A* c.1770 + 5 G > C was proved to disrupt the normal splicing of intron 13 and led to a premature stop codon at position 618 (p.(P591Vfs*28)). *POLR1C*, identified in 2015 [24], is a relatively new disease-causing gene of HLDs. According to the HGMD database, only 16 missense mutations and 6 small deletions have been reported so far. It is also the first report of mutations in *POLR1C* in Chinese HLDs patients.

Of note, *SOX10* is not a classical HLDs gene, and heterozygous variants in *SOX10* lead to a very wide spectrum of phenotypes including peripheral demyelinating neuropathy, central dysmyelination, Waardenburg syndrome, Hirschsprung disease, Kallmann syndrome [25], and deafness. Although 78 intra-genic variants in *SOX10* have been reported (data from LOVD database), no genotype-phenotype relationship can be defined yet. Our case (Pt7) presented with mild developmental delay and diffuse hypomyelination without the involvement of other systems, which may suggest another form of *SOX10* related disorder.

It is also worth noting that, of all six previously known genes with causative variants, *TUBB4A, POLR3A*, and *SOX10* were included in our gene list of targeted NGS performed previously. However, the variants were missed when they were evaluated. This suggests the necessity of periodic data reanalysis with improved bioinformatics. The gene *POLR1C* has been identified recently and was not included in the targeted NGS panel. This emphasizes the indispensability of updating the gene list of the panel over time. Moreover, it also demonstrates the advantage of WES in comprehensive coverage of all genes compared to the gene panel.

From the inheritance pattern or origin of variants perspective, it is outstanding that, of 15 variants, seven arose de novo. Although the rate might be overestimated due to the small number of the cohort, it is much higher than we expected. Out of 19 HLDs listed in the OMIM database currently, dominant inheritance is only present in three sub-type HLDs, HLD6, HLD16, and HLD19, which are caused by a heterozygous variant in *TUBB4A*, *TMEM106B,* and *TMEM63A*, respectively. All three genes were identified by trio-based WES in sporadic cases in recent years [9, 11, 26]. In our prior 155 HLDs patients with definite genetic diagnosis, except eight patients with de novo variants in *TUBB4A*, all the remaining 94.8% of patients (147/155) were diagnosed with the recessive disorder (119 cases have been published [4]). Many cases with HLDs have a fully penetrant, severe disorder preventing procreation, such that dominant inheritance cannot be observed. This explains the preponderance of de novo heterozygous variants in this and the prior collections of HLDs cases. On the other hand, in previous studies, the identification of novel genes depended on the application of linkage analysis followed by Sanger sequencing of candidate genes in consanguineous families or big pedigrees with multiple affected individuals, and sporadic cases with de novo variants were omitted. WES of parent–child trios is a highly effective approach for identifying de novo variants [27] and will help discover more genes with heterozygous variants in HLDs.

In conclusion, through trio-based WES, 11 of 20 HLDs patients were genetically diagnosed with seven different Mendelian disorders. This analysis emphasizes the heterogenicity of HLDs, the diagnostic power of trio-based WES for HLDs, and novel mutations identified in *TUBB4A, POLR3A*, and *POLR1C*. Moreover, comprehensive analysis including a focus on candidate genes helps to discover novel disease-causing genes, determine the diagnosis for the first time, and improve the yield of WES.
